# Alcohol Affects Neuronal Substrates of Response Inhibition but Not of Perceptual Processing of Stimuli Signalling a Stop Response

**DOI:** 10.1371/journal.pone.0076649

**Published:** 2013-09-25

**Authors:** Kyriaki Nikolaou, Hugo Critchley, Theodora Duka

**Affiliations:** 1 Behavioural and Clinical Neuroscience, School of Psychology, University of Sussex, Brighton, United Kingdom; 2 Clinical Imaging Science Centre, Brighton and Sussex Medical School, Brighton, United Kingdom; 3 Sackler Centre for Consciousness Science, University of Sussex, Brighton, United Kingdom; 4 Sussex Partnership NHS Foundation, Brighton and Hove, United Kingdom; The University of Queensland, Australia

## Abstract

Alcohol impairs inhibitory control, including the ability to terminate an initiated action. While there is increasing knowledge about neural mechanisms involved in response inhibition, the level at which alcohol impairs such mechanisms remains poorly understood. Thirty-nine healthy social drinkers received either 0.4g/kg or 0.8g/kg of alcohol, or placebo, and performed two variants of a Visual Stop-signal task during acquisition of functional magnetic resonance imaging (fMRI) data. The two task variants differed only in their instructions: in the classic variant (VSST), participants inhibited their response to a “Go-stimulus” when it was followed by a “Stop-stimulus”. In the control variant (VSST_C), participants responded to the “Go-stimulus” *even if* it was followed by a “Stop-stimulus”. Comparison of successful Stop-trials (Sstop)>Go, and unsuccessful Stop-trials (Ustop)>Sstop between the three beverage groups enabled the identification of alcohol effects on functional neural circuits supporting inhibitory behaviour and error processing. Alcohol impaired inhibitory control as measured by the Stop-signal reaction time, but did not affect other aspects of VSST performance, nor performance on the VSST_C. The low alcohol dose evoked changes in neural activity within prefrontal, temporal, occipital and motor cortices. The high alcohol dose evoked changes in activity in areas affected by the low dose but importantly induced changes in activity within subcortical centres including the globus pallidus and thalamus. Alcohol did not affect neural correlates of perceptual processing of infrequent cues, as revealed by conjunction analyses of VSST and VSST_C tasks. Alcohol ingestion compromises the inhibitory control of action by modulating cortical regions supporting attentional, sensorimotor and action-planning processes. At higher doses the impact of alcohol also extends to affect subcortical nodes of fronto-basal ganglia- thalamo-cortical motor circuits. In contrast, alcohol appears to have little impact on the early visual processing of infrequent perceptual cues. These observations clarify clinically-important effects of alcohol on behaviour.

## Introduction

A weakening of inhibitory control (i.e. the ability to withhold prepotent — automatic or motivated — behavioural responses) is implicated in the development of alcohol addiction [1]. In adolescents, the pattern of neuronal activity associated with performing tasks that require increased control of behaviour, can predict vulnerability to alcohol abuse [2]. Moreover, a lack of behavioural control over drinking is considered to be both a risk factor to alcohol abuse, and a secondary consequence of excessive drinking [3]. Alcohol intoxication impairs the general ability to inhibit inappropriate, or context irrelevant behaviours, evidenced by increased aggressiveness and risky driving [4], and it may contribute to binge drinking. However, the neural mechanisms by which acute alcohol affects inhibitory control are poorly understood. Examining the brain mechanisms involved in the effects of alcohol on inhibitory control will contribute more generally to our understanding of alcohol abuse.

In the laboratory, both, the Stop-signal task (SST) [5], which tests the ability to stop an initiated response when a Stop-signal appears, and the Go/No-Go task, which tests the ability to withhold a dominant pre-potent response, have been used to examine the effects of acute alcohol ingestion on the ability to inhibit behavioural responses [6,7].

Behavioural studies typically demonstrate impairments following the acute administration of alcohol in these measures of inhibitory control [but see 8,9]. Specifically, acute alcohol, at doses ranging between 0.45g/kg and 0.65g/kg, increased the number of failed inhibitions on a cued Go/No-Go task [7,10,11,12,13], and on an auditory SST [14,15]; [6,16,17,18]. In a visual SST, acute alcohol at doses between 0.62g/kg and 0.8g/kg impaired inhibitory control, as evidenced by a significant increase in Stop-signal reaction time (SSRT; an index of the speed of the stopping process) [19,20].

Neuroimaging (e.g. functional magnetic imaging; fMRI) studies in healthy volunteers performing SST and Go-No/Go tasks demonstrate the importance of a fronto-basal-ganglia network, encompassing the right inferior frontal gyrus (IFG; the pars opercularis moving into the insula), the dorsolateral prefrontal cortex (middle frontal gyrus), the medial prefrontal cortex (pre-supplementary motor area; pre-SMA) and the basal ganglia/thalamus, in response inhibition [21,22,23,24,25,26]. The engagement of these regions during inhibition tasks is consistent with their role in cortico-basal ganglia-thalamo-cortical motor loops [27,28].

A prevalent conceptualization of behavioural inhibition, proposes that the functional processes underlying “Go” and “Stop” responses are largely independent. Excitatory motor processes activated by the Go-signal compete in time with inhibitory processes triggered by the Stop-signal. Upon detection of the Stop-signal, prefrontal cortical regions (IFG or pre-SMA) initiate a counter-command that is relayed to the basal ganglia, where functional nodes associated with “Going” are suppressed in favour of those associated with “Stopping”. Subsequent output from the basal ganglia via global pallidus to the thalamus, blocks the thalamic “Go” signal to motor cortices, and the initiated motoric response is terminated [23,25,29,30,31]. The relay of the “Stop” information from the cortex to the thalamus has been found to engage the “indirect” (cortex – striatum – global pallidus external – global pallidus internal – thalamus - cortex) and the “hyperdirect” (cortex – subthalamic nucleus – global pallidus internal – thalamus - cortex) cortico-basal-anglia-thalamocortical loops [30,32,33].

Recent fMRI studies [34,35,36,37,38,39] have helped delineate distinct roles for the IFG and pre-SMA in SST and Go/No-Go task performance: The IFG is activated during the control of motor responses and successful stopping, but also more generically in response to presentations of infrequent visually-salient stimuli, and when shifting attention between cues. In contrast, the pre-SMA is more directly implicated in inhibition of motor action.

Ιntegral components of the ability to inhibit a behavioural response include processes involved in performance monitoring, and more specifically, the ability to process failed inhibitions (i.e. errors) in SST and Go-No/Go tasks. The region most commonly associated with these functions is the dorsal anterior cingulate cortex (ACC) [40,41].

To date, only two published fMRI studies have assessed the effect of acute alcohol ingestion on regional brain responses during Go-No/Go and SST task performance [42,43]. Specifically, Anderson and colleagues [42] administered two doses of acute alcohol (breath alcohol levels of 0.05% and 0.10%) and tested healthy social drinkers’ performance on a Go-No/Go task. Behaviorally, they observed a dose-dependent increase in the number of incorrect No-Go trials. At the higher dose of alcohol, there were decreases in activity within bilateral precentral cortex, pre-SMA, thalamus, insula, anterior cingulate cortex, postcentral gyrus, temporal lobe, and bilateral inferior orbitofrontal cortex during incorrect No-Go trials. However, lack of an assessment of regional neural activity associated with successful No-Go trials, limits direct inferences about the effects of acute alcohol on the stopping process *per se*.

The study of Schuckit and colleagues [43] examined the effects of 0.7ml/kg of alcohol during an SST in groups of social drinkers with high and low levels of response to acute alcohol. The SST that Schuckit and colleagues used did not vary Stimulus Onset Asynchrony (SOA) according to the typical staircase procedure that ensures successful inhibition of response on 50% of Stop-trials, but it did include individualized “easy” (the Stop-signal occurred earlier in time than participants’ mean Go reaction time) and “difficult” (the Stop-signal occurred at or near participants’ mean Go reaction time) to inhibit Stop-trials. Consequently, differences in inhibitory performance were reflected in accuracy measurements, and in the proportion of successful “easy” and “difficult” Stop-trials. In addition, due to the focus of the paper, the authors do not present data associated with the effects of acute alcohol across the two groups of participants. Nevertheless, in those individuals with a low-response to alcohol (most at risk of problem drinking), alcohol suppressed responses within temporal and parietal lobe regions when there was a failure to inhibit responses to a Stop-signal. Moreover these effects were amplified and extended to prefrontal and anterior cingulate regions for those trials where inhibition was most difficult.

Thus based on the evidence described so far, acute alcohol leads to measurable behavioural changes on established measures of response inhibition. However, the neural mechanisms by which alcohol compromises response inhibition remain incompletely understood. Possibilities include the direct influence of alcohol on fronto-striatal pathways that normally inhibit inappropriate actions, either by affecting sites within the basal ganglia, or prefrontal components of these networks. Another possibility is that acute alcohol ingestion impairs the early attention-dependent perceptual processing of infrequent Stop-signals that would normally elicit a re-evaluation of behavior, and consequently the inhibition of an inappropriate action: Alcohol in moderate doses can influence the processing of salient and infrequent stimuli (Stop-signals represent both) within visual cortical regions [44,45]. Additionally, alcohol can enhance spontaneous fluctuations in haemodynamic (blood oxygenation level-dependent; BOLD) signal within primary visual cortex, an effect that might plausibly impair neural responses to, and perception of, visual stimuli [46].

Consequently, the aim of the current study was to examine the neural mechanisms by which alcohol influences motor inhibition (stopping processes) using the SST. We administered two different doses of alcohol (0.4g/kg and 0.8g/kg) to test for dose-response relationships in alcohol’s effects. Furthermore, we employed a visual SST (VSST), in which the Go and Stop stimuli were identical in every respect except in colour, and which utilized a performance-dependent staircase procedure to modulate SOA: The SOA is titrated through incremental increases or decreases, respectively, after correct or incorrect inhibition trials. At shorter SOAs, stopping is easier than at longer SOAs. The staircase procedure ensured that participants were able to successfully inhibit their response on 50% of Stop-trials, and allowed for the calculation of the SSRT, by subtracting the mean SOA from the mean Go-latency. The staircase procedure also ensured that participants would not differ in the number of successful and unsuccessful Stop-trials. However, if alcohol had an effect, participants would differ in the speed of the stopping process (i.e. SSRT), thereby revealing how effective the stopping mechanism is as a function of acute alcohol intoxication.

We also employed a control variant of the SST, in which participants were told to continue to respond irrespective of whether the Go-stimulus was followed by a Stop-stimulus. In this control variant (VSST_C), the Stop-signals occurred as frequently as in the typical variant and were perceptually identical to the Stop-signals of the typical variant. The inclusion of this control task allowed us to test whether the effects of alcohol on the SST are mediated by an effect on the mechanisms responsible for processing an infrequent, visually salient stimulus in general.

In line with a previous study that examined the effect of alcohol on response inhibition [43], we additionally explored the effect of the two doses on error processing, by examining activations associated with unsuccessful Stop-trials relative to successful Stop-trials.

We predicted that behaviourally, alcohol would impair inhibitory control as evidenced by an increase in SSRT. In addition, we predicted that this effect of alcohol would be mediated by an effect on components of fronto-striatal networks implicated in the inhibition of an initiated motoric response. If however this effect of alcohol was also mediated by an impairment in the processing of infrequent and perceptually salient stimuli, this would be revealed through fMRI conjunction analyses with the control SST variant. Consistent with evidence from previous studies, alcohol effects on error processing were predicted to be revealed through changes in the engagement of anterior cingulate cortex.

## Materials and Methods

### Participants

The study recruited 42 moderate-to-heavy social drinkers (21 male and 21 female) from the University of Sussex subject pool. Advertisements requested right-handed, English-speaking social drinkers, between 18 and 40 years of age. Inclusion to the study required a weekly alcohol consumption of 10-60 units, as assessed using the Alcohol Use Questionnaire (AUQ; One unit is equivalent to 8g of ethanol, and refers to a small glass of wine, half-a-pint of beer, or a shot of spirits straight or mixed) [47]. Exclusion criteria included: a history of psychiatric problems, high blood pressure, or any other cardiovascular condition; regular cannabis use; smoking more than 20 cigarettes/day; pregnancy, attempting to conceive, or breastfeeding; or the use of any medication for any psychological or physical condition at the time of the study [excluding contraceptives, but including antibiotics and paracetamol (acetaminophen)]. Further exclusion criteria included having metal implants, teeth braces or bridges, tattoos above the shoulder, or a cardiac pacemaker. Written informed consent was provided by all participants, and the study was approved by the University of Sussex ethics committee. Participants were reimbursed in cash or in course-credits for their participation.

### Design/Procedure

Participants were randomly allocated to receive one of three flavoured drinks that contained either a low dose of ethanol (0.4g/kg of body weight; Low-dose group), a high dose of ethanol (0.8g/kg of body weight; High-dose group), or no ethanol (Placebo group), under double-blind conditions.

Participants took part in an initial baseline/task-familiarization session and a subsequent drink-administration/scanning session (see below for details). During scanning, participants completed two variants of a Visual Stop-signal Task (VSST) alongside other tasks. The two VSST variants were always presented 20-25 minutes after drink administration, when the blood alcohol concentration reaches a plateau [48].

Prior to each session, participants were asked to refrain from drinking alcohol- or caffeine-containing drinks for at least 12 and 4 hours, respectively, and from taking illicit drugs for one week. They were also asked to refrain from consuming a high-fat-content breakfast or lunch prior to testing. Participants were allowed to smoke as they would normally before each session, but smoking was not permitted during testing.

A standard breathalyser (Lion Alcolmeter SD-400; Lion Laboratories Ltd, Barry, UK), with a detection-limit equivalent to 0.01 g/l of alcohol in the bloodstream, was used to measure breath alcohol concentrations (BRaCs) at the start of each session to ensure zero blood alcohol levels. BRaCs were converted to blood alcohol concentrations (BACs), and are reported as BACs throughout the paper, as is standard for alcohol-related studies.

Three days before scanning, participants gave fully-informed consent to take part in the study, and provided demographic details (age, gender and weight) and information regarding their medical history (assessed using the Nuffield Hospitals Medical History Questionnaire which covers past and present physical and psychiatric health status, including any current medication), including estimates of the number of cigarettes they smoked per day. They also completed a number of measures that were used to ensure that the three groups were matched on baseline cognitive ability (Rey Auditory Verbal Learning Test; RAVLT) [49], on baseline facets of trait impulsivity (Barratt Impulsiveness Scale; BIS) [50], as well as in their drug-use history (Drug Use history Questionnaire; DUQ) [51], their average weekly alcohol-use (Use Questionnaire; AUQ) [47], and their baseline expectations concerning the effects of alcohol (Alcohol Expectancy Questionnaire; AEQ) [52]. Participants also completed a baseline run of the VSST (see below).

During the main scanning session, participants were given their allotted drink and then were placed in the 1.5Tesla Siemens Avanto MRI scanner where they completed the variants of the VSST during acquisition of functional (fMRI) datasets. Subjective feelings associated with alcohol ingestion (Subjective Effects Visual Analogue Scale; VAS) [53] were rated immediately before and 10 minutes after drink administration. BACs were also taken at these times, and later after completion of the scanning procedure. Each participant was also asked to guess the type of drink they thought they had received, and to rate how confident they were about their guess on a 4-point scale ranging from 1 (“not at all confident”) to 4 (“very confident”). At the end of the main scanning session, participants were debriefed, and those from the alcohol groups remained in the laboratory until their BACs had fallen below 0.4 g/l (half of the legal driving limit).

#### Alcohol preparation/administration

Alcohol was administered at a dose of either 0.4g/kg or 0.8g/kg of body weight. Each dose was diluted with tonic water (Schweppes, Uxbridge, UK) to create a 500ml drink. The placebo beverage consisted only of the respective volume of tonic water (i.e. 500ml). Six drops of Angostura bitters® were added to the placebo and the alcohol beverages to match the taste of each drink-type, and to disguise the taste of the alcohol. Each drink-type was administered in 10×50ml portions and was consumed at a rate of one portion every 3 minutes. Drinks were prepared by another researcher to ensure the double-blind conditions, and the experimenter was present throughout the drinking procedure.

### Measures

#### Trait characteristics

The following measures were administered in the initial familiarisation session, and were used to ensure that the groups were matched on baseline trait impulsivity and cognitive ability, as well as in their drug-use history, their average weekly alcohol-use, and their baseline expectations concerning the effects of alcohol.

The Rey Auditory Verbal Learning Test (RAVLT) [49] is a working memory (WM) capacity index. A list of 10 words is read-out at a rate of one word every 2 seconds. Following a delay of 2 minutes, participants are asked to repeat back as many words as they can remember. High scores index high WM capacity.

In the *Drug Use history Questionnaire* (DUQ) [51], participants state whether they have ever used any of the drugs listed in the questionnaire (drugs derived from the main drug categories, e.g. marijuana, cocaine, ecstasy etc.), and provide an estimate of the frequency of use, and the usual dose consumed per session.

The Alcohol Use Questionnaire (AUQ) [47] gives an estimate of the average number of weekly alcohol-units consumed over the previous 6 months (a glass of wine is measured as 1.5 units; a pint of beer/cider as 2.4 units; a shot of spirit as 1 unit; and a bottle of alcopops as 1.7 units). An overall score is also calculated based on the weekly alcohol-unit consumption, the speed of drinking (number of drinks per hour), the number of episodes of alcohol intoxication in the past 6 months, and the percentage of alcohol intoxications out of the total number of times of going out drinking.

The Alcohol Expectancy Questionnaire (AEQ) [52] is a 38-item measure based on Fromme’s Comprehensive Effects of Alcohol Questionnaire [52], and assesses expectations concerning the effects of alcohol. It consists of seven factors, of which four factors form the “Positive” (factors 1-4: sociability, tension reduction, liquid courage and sexuality), and three form the “Negative” (factors 5-7: cognitive and behavioural impairments, risk and aggression, and negative self-perception) expectancies. High scores on the “Positive” and the “Negative” factors is indicative of expecting increased positive and increased negative outcomes from the consumption of alcohol, respectively.

The Barratt Impulsiveness Scale (BIS) [50] is a 30-item questionnaire designed to measure three aspects of impulsivity: (a) non-planning impulsivity or the inability to plan and think carefully; (b) motor impulsivity or acting on the spur of the moment; and (c) attentional impulsivity or the inability to focus on the task at hand. Items are rated on a 4 point Likert-type scale ranging from “rarely/never” to “almost always”. Higher scores represent greater levels of impulsive behaviour.

#### Subjective Effects Visual Analogue Scale (VAS) [53]

A self-report Likert-type scale (100mm long) was used to assess changes in subjective feelings associated with alcohol ingestion. The scale included an estimation of feeling light headed, irritable, stimulated, alert, relaxed, and contented (poles: not at all–very much).

#### Visual Stop-signal Task (VSST)

Two versions of the VSST were used, modelled on the work by Boehler and colleagues and Sharp and colleagues [34,35,39]. The two versions differed only in the instructions given to participants. In the more classic variant of the task (VSST), participants were instructed to respond to the direction of a frequently occurring green arrow (Go-stimulus) as quickly and as accurately as possible, and to be as accurate as possible at withholding their response when the green arrow turned red (Stop-stimulus). In the “Control” (VSST_C) variant, participants were told to respond to the direction of the Go-stimulus, again as quickly and as accurately as possible, but to carry on, and not withhold their response if it was followed by a Stop-stimulus.

**Figure 1:**
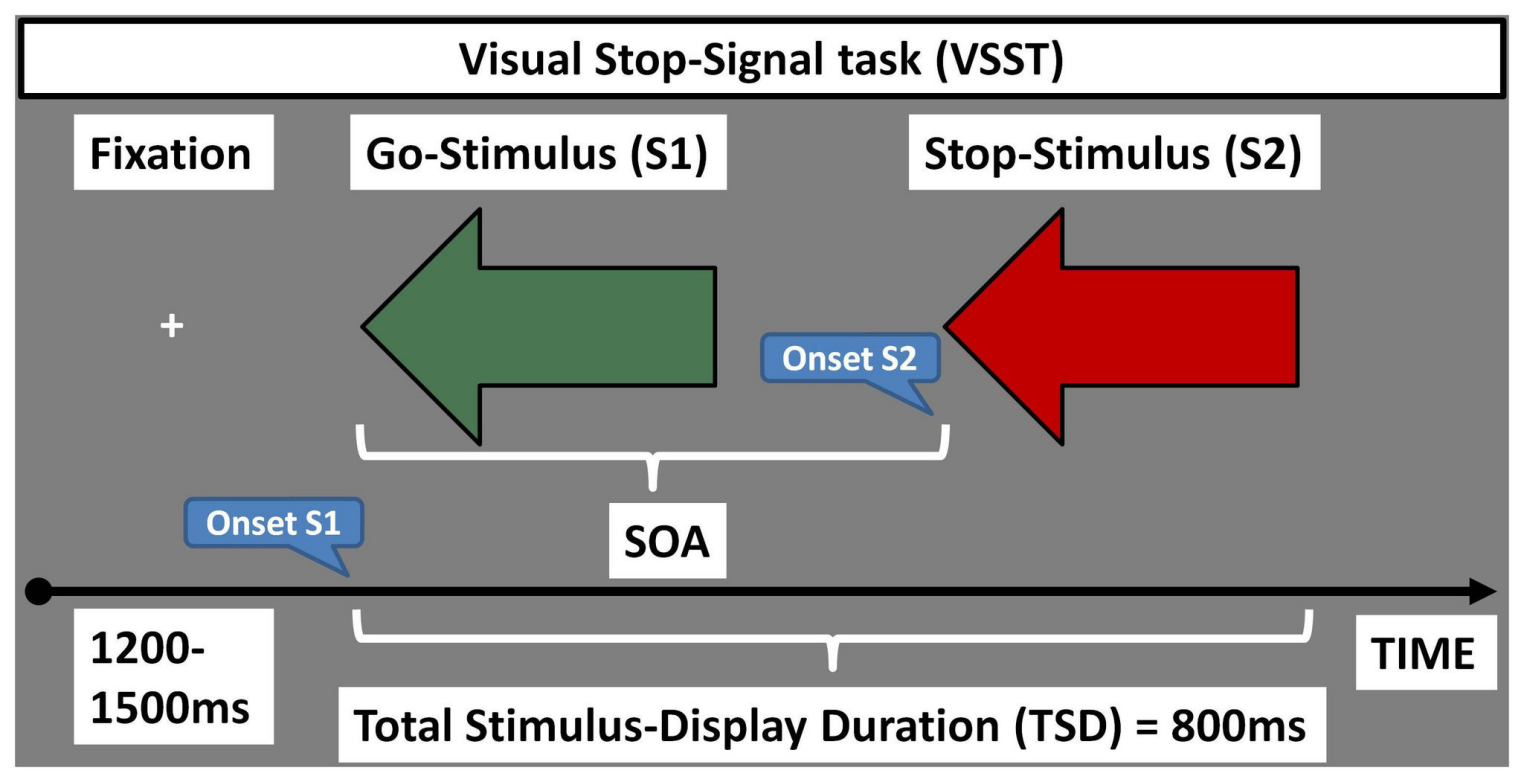
Visual Stop-signal task (VSST): Participants responded to the direction of a central green arrow (“Go-stimulus”), and withheld their response when the arrow turned red (“Stop-stimulus”) in the classic variant of the task. In the control variant, participants were told to respond as they would normally to the direction of the “Go-stimulus” irrespective of whether it was followed by a “Stop-stimulus”. In both variants of the VSST, each trial began with the presentation of a fixation cross of varied duration. On “Stop-trials”, the “Go-stimulus” was replaced, following a varied Stimulus Onset Asynchrony (SOA), by the “Stop-stimulus”. In the classic variant, the SOA increased or decreased by 50msec as a function of whether participants were correct or not, respectively, at inhibiting their response to the “Stop-stimulus”. In the control variant, the SOA also varied in 50msec increments, but randomly. The total stimulus-display duration (TSD) was 800ms.

In both variants, each trial began with the presentation of a central fixation cross on a grey background for a jittered duration of 1200-1500ms. The stimulus-display that followed, always began with the presentation of the Go-stimulus on a grey background, which either remained on screen for a total stimulus-display duration (TSD) of 800ms (Go-trials), or was replaced, after a variable stimulus onset asynchrony (SOA), by the Stop-stimulus (Stop-trials; see Figure 1).

In the VSST, the initial SOA was 200ms and increased by 50ms following successful Stop-trials (i.e. Stop-trials in which participants successfully inhibited their response; SStop), or decreased by 50ms following unsuccessful Stop-trials (i.e. Stop-trials in which participants were not able to inhibit their response; UStop). This staircase procedure resulted in an even number of SStops and UStops [54]. Stop-signal reaction time (SSRT) was calculated by subtracting the mean SOA from the average reaction time to correct Go-trials [55]. Further dependent measures from the VSST included Go-latency (Go_LAT) and Go-accuracy (Go_ACC). Participants completed a total of 160 trials of the VSST (120 Go-trials and 40 Stop-trials) in 8 minutes.

For each participant, in each group, the VSST was always followed by the VSST_C. Here, the initial SOA was the SOA-value from the final VSST Stop-trial. In order to equate the two variants, the SOA of the VSST_C Stop-trials also varied by 50ms steps, but in a random one-up/one-down fashion. Dependent measures included Go-latency (Go_LAT_C), Go-accuracy (Go_ACC_C), Stop-latency (Stop_LAT_C) and Stop-accuracy (Stop_ACC_C). Participants completed a total of 80 trials of the VSST_C (60 Go-trials and 20 Stop-trials) in 4 minutes.

In the initial baseline/task familiarisation session participants completed a baseline run of the classic VSST in order to make sure that the groups were matched on VSST performance prior to the alcohol manipulation.

### fMRI Methods

fMRI was performed on a 1.5Tesla Siemens Avanto MRI scanner (Siemens, Erlangen, Germany (quadrature birdcage transceiver headcoil). T2*-weighted images echoplanar images sensitive to blood oxygenation level-dependent (BOLD) contrast were acquired, covering the entire head (36 slices, 3mm isotropic voxels, TR 3300ms, TE 50ms, 64x64 matrix). Slices were angled -30° in the anteroposterior axis to reduce susceptibility-induced BOLD sensitivity losses in orbitofrontal regions [56,57]. Functional data were acquired in one continuous session per VSST variant (160 volumes per subject for the VSST and 83 volumes per subject for the VSST_C; the initial 4 volumes were discarded to ensure steady state B0 magnetization).

Anatomical images of each subject’s brain were collected using a T1-weighted Magnetization-Prepared Rapid Acquisition Gradient Echo (MP-RAGE) sequence (56 X 256 matrix 0.9 mm isotropic voxels).

The fMRI data were preprocessed and statistically analyzed using SPM8 (Wellcome Trust Centre for Neuroimaging, University College London, UK) and MATLAB 7 (The MathWorks, Inc., Natick, MA, USA). Functional images were slice-time and motion corrected; spatially normalized to standard MNI (Montreal Neurological Institute) space [58]; re-sampled to 2mm isotropic voxels and; smoothed using an 8mm full-width at half-maximum Gaussian kernel.

### Exclusion of data from analyses

The data from 39 participants (19 male and 20 female; 13 participants in the placebo group, 13 in the low-dose group and 13 in the high-dose group) were analysed. Data from three participants were excluded from all analyses (behavioural and fMRI) either because the data were not recorded due to a technical fault (1 participant), or because the participant did not withhold his/her response on any VSST Stop-trial during scanning (2 participants).

### Analysis of behavioural/questionnaire data

#### Demographic and Trait characteristics

Differences between groups on demographic information and scores derived from the DUQ, the AUQ, the Positive/Negative factors of the AEQ, the non-planning/motor/attentional factors of the BIS, and the RAVLT, were examined using chi-square and one-way Analyses of Variance (ANOVA) as appropriate. Significant ANOVAs were explored further using Bonferroni-corrected post-hoc independent-samples t-Tests.

#### State characteristics and BACs

Subjective ratings from each factor derived from the VAS were analysed using mixed 3x2 ANOVAs with group (placebo vs. low-dose vs. high-dose) and time (pre- vs. post-drinking) as factors. Significant group x time interactions were explored further using post-hoc paired samples, Bonferroni-corrected t-Tests that tested pre- versus post-drinking differences in each group separately. Differences between male and female participants in BAC were tested using two separate mixed 2x2 ANOVAs, one in the low and one in the high-dose group, that included gender (male vs. female) and time (pre- vs. post-scanning) as factors.

#### VSST and VSST_C – Behavioural analyses

Group differences on VSST indices [i.e. SSRT, Go-accuracy (Go_ACC), and Go-latency (Go_LAT)] were tested using 3 mixed 2 x 3 ANOVAs with time (baseline vs. scanning session) as the within-subjects factor and group (placebo vs. low-dose vs. high-dose) as the between subjects factor. The factor time was included to ensure that groups did not differ on any VSST index at baseline. Significant time x group interactions were explored further using one-way ANOVAs comparing the performance of the three groups separately at baseline and during the main scanning session. Significant effects were explored further using post-hoc Bonferroni-corrected independent samples t-Tests.

In order to ensure that VSST_C Stop- and Go-trials did not differ on latency or accuracy scores, mixed 2x3 ANOVAs were undertaken with trial-type (go vs. stop) as the within-subjects factor and group (placebo vs. low-dose vs. high-dose) as the between subjects factor. Significant group x trial-type interactions were explored further using one-way ANOVAs comparing the performance of the three groups separately during Go-trials and during VSST_C Stop-trials. Significant effects were explored further using post-hoc Bonferroni-corrected independent samples t-Tests.

We also ran “*Between VSST-variant analyses*” in order to ensure that trials requiring a response (i.e. Go- and Stop-trials in the VSST_C and Go-trials in the VSST variant) did not differ with respect to latency or accuracy scores. Mixed 3x3 ANOVAs were undertaken with trial-type (Go_ VSST_C vs. Stop_VSST_C vs. Go_VSST) as the within-subjects factor and group (placebo vs. low-dose vs. high-dose) as the between subjects factor. Significant group x trial-type interactions were explored with one-way ANOVAs and post-hoc Bonferroni-corrected t-Tests.

### VSST and VSST_C – fMRI analyses

Task and condition-related changes in neural activity were derived from regional changes in BOLD contrast consequent upon evoked haemodynamic responses. For the analyses of the fMRI data, two independent statistical models, one per VSST variant, were initially computed for each participant in each group. For each model, condition-specific event onsets were convolved with the canonical haemodynamic response function and a high pass filter (128 s) was applied to remove low-frequency artefacts [59]. Events were modeled at the onset of the Go-stimuli.

Specifically, the VSST 1^st^ level models included event onsets of correct Go-trials (Go), successful Stop-trials (Sstop), and unsuccessful Stop-trials (Ustop). Movement regressors, incorrect Go-trials, as well as fixation onsets were included as regressors of no interest.

The control VSST 1^st^ level models included event onsets of correct Go- (Go_VSST_C), and of correct Stop-trials (Stop_VSST_C). Movement regressors, and incorrect Go/Stop trials, as well as fixation onsets were included as regressors of no interest.

We computed the contrasts Sstop>Go (VSST contrast), Ustop>Sstop (VSST-Error contrast), and Stop_VSST_C>Go VSST_C (VSST_C contrast) for each participant. These contrast images were submitted to separate second level random-effects analyses [60,61].

#### Task-related effects – Placebo group only

The VSST the VSST_C contrasts were submitted to two separate random effects analyses using one-sample t-Tests in the placebo group only, in order to examine regions of activation uncontaminated by the effects of alcohol.

The VSST_C thresholded SPM map was used as an exclusive mask to the VSST analysis in order to identify clusters that were NOT involved in the processing of infrequent Stop-stimuli.

In order to identify the cerebral substrates involved in the processing of infrequent Stop-stimuli over frequent Go-stimuli in general (i.e. irrespective of whether participants were asked to inhibit their response) a conjunction analysis of the VSST and the VSST_C one-sample SPMs was also performed. This conjunction tested for significant clusters that survived thresholding in *both* analyses [62].

#### Alcohol-related effects

The VSST contrast was submitted to a one-way ANOVA. The main effect of group F-statistic was computed to examine differences in BOLD activation between the three groups.

We were also interested in examining possible non-linear effects of the two alcohol doses. We therefore submitted the VSST contrast images to 3 separate two-sample t-Tests to examine differences in BOLD response between: (a) the placebo group and the low alcohol-dose group; (b) the placebo group and the high alcohol-dose group; and (c) the two alcohol doses. F contrasts were computed for each of these comparisons.

The VSST-Error contrast was submitted to a one-way ANOVA. The main effect of group F-statistic was computed to examine differences in BOLD activation between the three groups.

Non-linear effects of the two alcohol doses were assessed by submitting the VSST-Error contrast images to 3 separate two-sample t-Tests to examine differences in BOLD response between: (a) the placebo group and the low alcohol-dose group; (b) the placebo group and the high alcohol-dose group; and (c) the two alcohol doses. F contrasts were computed for each of these comparisons.

#### Regions of interest analysis to reveal the different mechanisms: VSST contrast

The coordinates of each significant cluster resulting from the VSST and the VSST_C contrast conjunction analysis in the placebo group (i.e. uncontaminated by any effects of alcohol) were used as centres of 4mm sphere Regions-of-Interest (ROIs). MarsBaR (http://marsbar.sourceforge.net/) was used to perform ROI analyses within the one-way ANOVA second-level model design. These analyses were thresholded at a family-wise-error corrected threshold of 0.05, given the restriction of the analysis within predefined regions.

#### One-way ANOVA: VSST_C contrast

We additionally examined the effects of acute alcohol on regions associated with just the processing of an infrequent signal, by entering the VSST_C contrast (i.e. Stop_VSST_C>Go VSST_C) into a one-way ANOVA.

#### Thresholding and localization

To protect against false-positive activations, unless otherwise stated, conjunction analyses, and all reported results met a threshold of p < 0.005 uncorrected, and a cluster volume exceeding 176mm^3^ (k = 13 voxels). This conjunction of specific voxel-level and cluster-extent thresholds corresponds to a whole-brain-corrected significance of p < 0.05. The non-arbitrary cluster-extent threshold (i.e. k = 13) was determined by Monte-Carlo simulation (62,1000 iterations; http://www2.bc.edu/_slotnics/scripts.htm) [63,64,65] to establish an appropriate voxel contiguity threshold [66], using the same parameters as in our study.

The anatomical localization of significant activations was assessed by superimposition of the SPM maps on the single-subject-T1-weighted MNI standard brain supplied by MRIcro (http://www.mccauslandcenter.sc.edu/mricro/index.html). Anatomical localization of subcortical regions was assessed using Duvernoy’s anatomical atlas [67].

## Results

### Demographic information, Trait characteristics and BAC

The three groups were well matched on all demographic/questionnaire/baseline indices (i.e. age, weight, DAQ, AUQ, BIS, RAVLT, and Positive/Negative AEQ scores: F < 2.3, ns, in all cases; and gender ratio, χ^2^ < 1, ns; See Table 1). The groups were also well matched in the number of smokers they contained (χ^2^ < 1, ns; all in all only eight smokers completed the study and were equally distributed in each group), as well as in the average number of cigarettes smoked daily (F < 1, ns).

**Table 1:** Demographic information (age, gender, weight), Trait characteristics (AUQ^**1**^, AEQ^**4**^, BIS^**3**^ and word recall), and BAC^**5**^ measurements (post-drinking and post-scanning) presented separately for the placebo, the 0.4g/kg, and the 0.8g/kg alcohol-dose groups.

**Variable**	**Placebo**	**0.4g/kg alcohol**	**0.8g/kg alcohol**
**Age (years)**	22.23 (±4.94)	22.08 (±3.38)	21.23 (±2.49)
**Gender**	6M, 7F	7M, 6F	6M, 7F
**Weight (kg)**	69.93 (±12.66)	68.18 (±12.76)	72.68 (±10.46)
**^1^AUQ – Weekly units**	25.24 (±14.67)	26.06 (±11.70)	27.95 (±10.07)
**AUQ - Total score**	46.26 (±25.67)	56.14 (±35.85)	52.60 (±25.51)
**Word Recall (RAVLT^2^ score)**	8.92 (±2.22)	8.54 (±1.81)	7.77 (±1.59)
**^3^BIS Attentional impulsivity**	16.62 (±3.52)	16.54 (±2.96)	18.31 (±3.15)
**BIS Motor impulsivity**	25.46 (±4.79)	22.85 (±3.02)	26.38 (±5.11)
**BIS Non-Planning**	24.31 (±4.57)	23.15 (±5.23)	25.46 (±4.84)
**^4^AEQ – Positive**	13.54 (±1.62)	13.46 (±1.86)	14.52 (±2.12)
**AEQ - Negative**	13.38 (±2.57)	14.26 (±1.93)	15.23 (±2.14)
**^5^BAC (g/l) -10 min Post Drink**	0	0.59 (±0.13)	1.16 (±0.34)
**BAC (g/l) -5 min Post Scanning**	0	0.35 (±0.09)	0.99 (±0.11)

**^1^AUQ (Alcohol Use Questionnaire**); **^2^RAVLT (Rey Auditory Verbal Learning Test**); **^3^BIS (Barratt Impulsiveness Scale**); **^4^AEQ (Alcohol Expectancy Questionnaire**); **^5^BAC (Blood Alcohol Concentration**)**; Data are presented in mean** (±**SD**)**.**

There were no gender differences with respect to post-drinking and post-scanning BACs in the high alcohol group (no main effect of gender, or gender x time interaction; Fs < 1.7, ns, in all cases). However, in the low-dose group, female participants had higher BACs than male participants both post-drinking and post-scanning (Main effect of gender: F(1, 11) = 6.77, p < 0.05; No gender x time interaction; F < 1, ns). As expected participants in the high alcohol dose group showed significantly higher BACs post-drinking, than those in the low-dose group [t(24)=5.53, p < 0.001]. Overall in the low-dose group, BACs ranged between 0.35g/l and 0.80g/l before scanning, while in the high-dose group they ranged between 0.80g/l and 2.19g/l before scanning (mean values are given in Table 1).

Blinding was successful in the placebo group, as only 4 participants (i.e. less than half) reported that they thought that they had received placebo. Interestingly, the majority of participants in the placebo group reported being somewhat confident that they had received the low alcohol dose. Participants in the two alcohol groups, however reported being “somewhat” to “very” confident that they had received alcohol, with only 2 participants in the low-dose group guessing placebo, and no participant in the high-dose group guessing placebo. Thus, while blinding was not successful in the two alcohol groups, probably due to the effects of alcohol itself, participants in the placebo group (i.e. the control group) were fully blind to the drink administration.

### VAS

Subjective reports of “lightheadedness” significantly increased following drink-administration in the placebo [t(12)=3.60, p < 0.005] and high-dose groups [t(12)=6.47, p < 0.001], but not in the low-dose group [t(12)=2.32, p = 0.04], explaining a significant time x group interaction F(2, 36) = 4.96, p < 0.05). There was a significant difference between groups pre-drinking [F(2, 36) = 4.96, p < 0.025] with the group allocated to the low alcohol dose showing the highest ratings. A significant time x group interaction (F(2, 36) = 3.73, p < 0.05] for the factor “contented” was due to that participants reported feeling more “content” following administration of the high alcohol dose [t(12)=2.32 p = 0.04], but not following administration of placebo or the low alcohol dose (t < 1, ns, in both cases). No other time x group interaction was statistically significant, and we did not find any main effects of group (Fs < 3.2, ns, in all cases). Mean VAS scores for each group pre- and post-drinking are presented in Table 2.

**Table 2:** Subjective ratings of feelings due to alcohol ingestion (VAS) taken pre- and post-drinking, presented separately for the placebo, the 0.4g/kg, and the 0.8g/kg alcohol-dose groups.

**VAS^1^ Factors**	**Time**	**Placebo**	**0.4g/kg alcohol**	**0.8g/kg alcohol**
**Light headed**	**Pre-drinking**	4.02 (±4.73)	27.69 (±32.14)	11.45 (±15.14)
	**Post-drinking**	29.83 (±24.70)	42.39 (±32.74)	55.98 (±27.06)
**Irritable**	**Pre-drinking**	3.68 (±4.12)	19.32 (±21.45)	11.97 (±15.34)
	**Post-drinking**	5.21 (±8.38)	6.84 (±7.89)	13.33 (±13.66)
**Stimulated**	**Pre-drinking**	53.59 (±20.53)	49.57 (±20.59)	50.09 (±13.57)
	**Post-drinking**	52.48 (±18.88)	50.43 (±17.26)	66.24 (±9.27)
**Alert**	**Pre-drinking**	60.60 (±14.29)	61.28 (±25.88)	51.45 (±17.89)
	**Post-drinking**	48.55 (±19.97)	42.65 (±18.02)	53.59 (±20.63)
**Relaxed**	**Pre-drinking**	62.82 (±17.80)	63.08 (±24.16)	56.75 (±20.98)
	**Post-drinking**	65.04 (±15.82)	71.54 (±18.69)	68.63 (±19.92)
**Contented**	**Pre-drinking**	63.33 (±16.23)	62.65 (±24.61)	56.41 (±18.28)
	**Post-drinking**	57.69 (±22.12)	65.21 (±21.13)	74.27 (±16.77)
**Pleasant glow**	**Pre-drinking**	50.94 (±19.91)	57.52 (±26.85)	51.20 (±22.77)
	**Post-drinking**	54.36 (±23.62)	71.20 (±21.64)	74.79 (±16.74)

1 **VAS (Subjective Effects, Visual Analogue Scale**)**; Data are presented in mean** **(±SD)** **; For significant effects see results-section.**

### VSST and VSST_C – Behavioural results

#### VSST

Stop-accuracy did not differ between alcohol groups in either the baseline or experimental session (F < 1, ns), and was approximately at 50%, suggesting that the staircase manipulation was effective.

There were no significant main effects of group, or time x group interactions with respect to Go-latency or Go-accuracy [Fs < 1, ns, in all cases]. However, we did observe a significant main effect of group in SSRT [F(2, 36) = 4.7, p < 0.05], with participants in the high-dose group displaying higher SSRTs overall relative to the placebo group [t(24) = 3.12, p < 0.01]. No other comparison survived Bonferroni adjustment [t < 2.3, ns, in both cases]. This main effect of group was qualified by a significant time x group interaction [F(2, 36) = 3.34, p < 0.05]. Groups did not differ in their baseline SSRT [F = 1.03, ns]. However, following drink- administration, both the low-dose and the high-dose group showed higher SSRTs when compared to the placebo group [t(24) > 2.47, p < 0.025, in both cases]. The two alcohol groups did not differ in SSRT [t(24) = 1.3, ns]. Mean indices derived from the VSST for each group are presented in Table 3.

**Table 3:** Indices of performance in the VSST (at baseline and during scanning) and VSST_C, presented separately for the placebo, the 0.4g/kg, and the 0.8g/kg alcohol-dose groups.

**Performance Index**	**Session**	**Placebo**	**0.4g/kg alcohol**	**0.8g/kg alcohol**
**VSST_SSRT^1^**	**Baseline**	257.21 (±25.31)	273.66 (±40.99)	278.32 (±48.55)
	**Scanning**	241.84 (±36.82)	279.95 (±41.44)	300.86 (±40.45)
**VSST_Go_Latency**	**Baseline**	519.90 (±87.59)	525.97 (±115.50)	528.35 (±84.84)
	**Scanning**	497.60 (±88.13)	505.43 (±92.06)	516.82 (±91.55)
**VSST_Go_Accuracy**	**Baseline**	98.78 (±1.11)	99.10 (±1.20)	98.85 (±1.30)
	**Scanning**	85.32 (±8.75)	94.55 (±8.07)	94.87 (±7.10)
**VSST_Stop_Accuracy**	**Baseline**	51.35 (±3.88)	56.35 (±7.50)	53.85 (±4.88)
	**Scanning**	51.73 (±3.44)	51.92 (±2.34)	48.46 (±3.26)
**VSST_C _Go_Latency**		411.20 (±55.77)	439.82 (±75.04)	444.32 (±64.92)
**VSST_C _Go_Accuracy**		98.46 (±1.44)	91.41 (±12.6)	97.18 (±2.30)
**VSST_C_Stop_Latency**		410.24 (±34.42)	437.34 (±87.63)	462.17 (±70.73)
**VSST_C_Stop_Accuracy**		91.67 (±17.00)	88.08 (±14.07)	95.42 (±4.77)

1 **SSRT (Stop-signal Reaction Time)** **; Data are presented in mean** **(±SD)** **; For significant effects see results-section.**

#### VSST_C

We found no main effects for trial-type or group, nor trial-type x group interactions with respect to either accuracy or latency (F < 3.6, ns, in all cases). Mean indices derived from the VSST_C for each group are presented in Table 3.

#### Between VSST variant analyses

We found no main effects of group, or significant trial-type x group interactions with respect to either accuracy or latency (F < 1.4, ns, in all cases). In addition, accuracy scores were equal across the three trial-types (i.e. no main effect of trial type for accuracy scores, F < 2.8, ns). However, Go-trials in the VSST were significantly slower than both Go- and Stop- trials in the VSST_C variant [Main effect of trial-type for latency scores, F(2, 48) = 29.2, p<0.001].

### VSST and VSST_C – fMRI results

#### Task-related effects – Placebo group only

Successful stopping relative to successful going (Sstop>Go) was associated with increases in activity within a set of regions including parts of the dorsolateral PFC, the inferior frontal gyrus pars opercularis, medial PFC and pre_SMA, the insula, and sectors within the basal ganglia. When the Sstop>Go contrast was masked exclusively with the Stop_VSST_C>Go VSST_C contrast, clusters within medial PFC, including pre-SMA and dorsolateral PFC (middle frontal cluster), basal ganglia and insular cortex were unaffected, suggesting that these regions were not merely involved in the perceptual processing of infrequent visual cues (see Table 4). Several other clusters were nevertheless affected by the exclusive masking, including the inferior frontal gyrus pars opercularis and parietal and temporal clusters (data not shown). However, a cluster within the inferior frontal gyrus pars opercularis survived exclusive masking (coordinates of this cluster are given in Table 4). Conjunction analyses between Sstop>Go and Stop_VSST_C>Go VSST_C contrast showed that specific clusters within the inferior frontal gyrus pars opercularis and within the parietal and temporal gyri (Table 5) were activated by the processing of infrequent, visually salient stimuli (red arrows) relative to frequent stimuli (green arrows), irrespective of whether these required the inhibition of a behavioural response.

**Table 4:** Sstop>Go activations that were unaffected by exclusive masking with the Stop_VSST_C>Go_VSST_C contrast in the placebo group (p <.005, unc. k ≥ 13 voxels).

**Region**	**Cluster**	**L/R**	**T**	**MNI coord. x, y, z**
***Prefrontal Cortex***				
Inferior Frontal pars Opercularis (BA48)	442	R	8.89	46 12 12 (32 14 12**^1^**)
Middle Frontal gyrus (BA46)	127	L	5.89	-42 38 34
Posterior insula (BA48)	15	R	4.21	36 -18 20
Anterior insula (BA48)	41	L	4.01	-28 26 4
Insula (BA48)	64	R	6.51	38 -2 -6
Pre-SMA (BA6)	294	R	5.53	12 12 48
Paracentral lobule/SMA proper (BA4)	17	L	4.62	-12-24 52
Middle cingulate	57	R	4.2	14-22 40
Precentral gyrus (BA6)	58	L	5.57	-30-6 48
***Parietal/Temporal cortex***				
Supramarginal gyrus (BA40)	107	L	4.82	-62-30 30
Superior Parietal gyrus	96	L	4.24	-40-48 62
Inferior Temporal gyrus	16	R	4.37	50-58 -18
Middle Temporal pole/Parahip/pal gyrus	112	R	7.43	22 6-32
Middle Temporal gyrus (BA37)	44	L	4.84	-58-64 4
***Occipital cortex/Cerebellum***				
Middle Occipital gyrus (BA18)	78	R	6.73	40-84 2
Cerebellum	90	L	5.61	-8-80 -44
***Subcortical areas***				
Putamen	26	L	4.43	-24 12 6
Caudate	19	L	4.3	-6 8 10
Hypothalamus	15	L	4.09	-8-8 -8

**BA = Broadmann area; R=right, L=left; MNI coordinates represent cluster peaks; ^**1**^The MNI coordinates for a sub-cluster within Frontal Pars Opercularis (BA48) which survived exclusive masking and was also affected by the low alcohol dose.**

**Table 5:** Activations resulting from conjunction analysis of Sstop>Go and Stop_VSST_C>Go_VSST_C contrasts in the placebo group (p <.005, unc. k ≥ 13 voxels).

**Region**	**Cluster**	**L/R**	**T**	**MNI coord. x, y, z**
***Prefrontal Cortex***				
Inferior Frontal pars Opercularis (BA48)	36	R	7.15	50 14 10
***Parietal/Temporal cortex***				
Supramarginal gyrus (BA40)	90	R	5.49	50 -42 40
Angular gyrus	79	R	5.42	44-58 50
Inferior Parietal gyrus	30	L	4.79	-52-48 42
Superior Temporal gyrus	15	R	4.27	50 -40 22
Middle Temporal gyrus	25	R	4.22	58 -50 2

**BA =** **Broadmann** **area** **; R=right,** **L=left;** **MNI** **coordinates** **represent** **cluster** **peaks.**

#### Alcohol effects in the VSST: One-way ANOVA: VSST contrast

**Figure 2:**
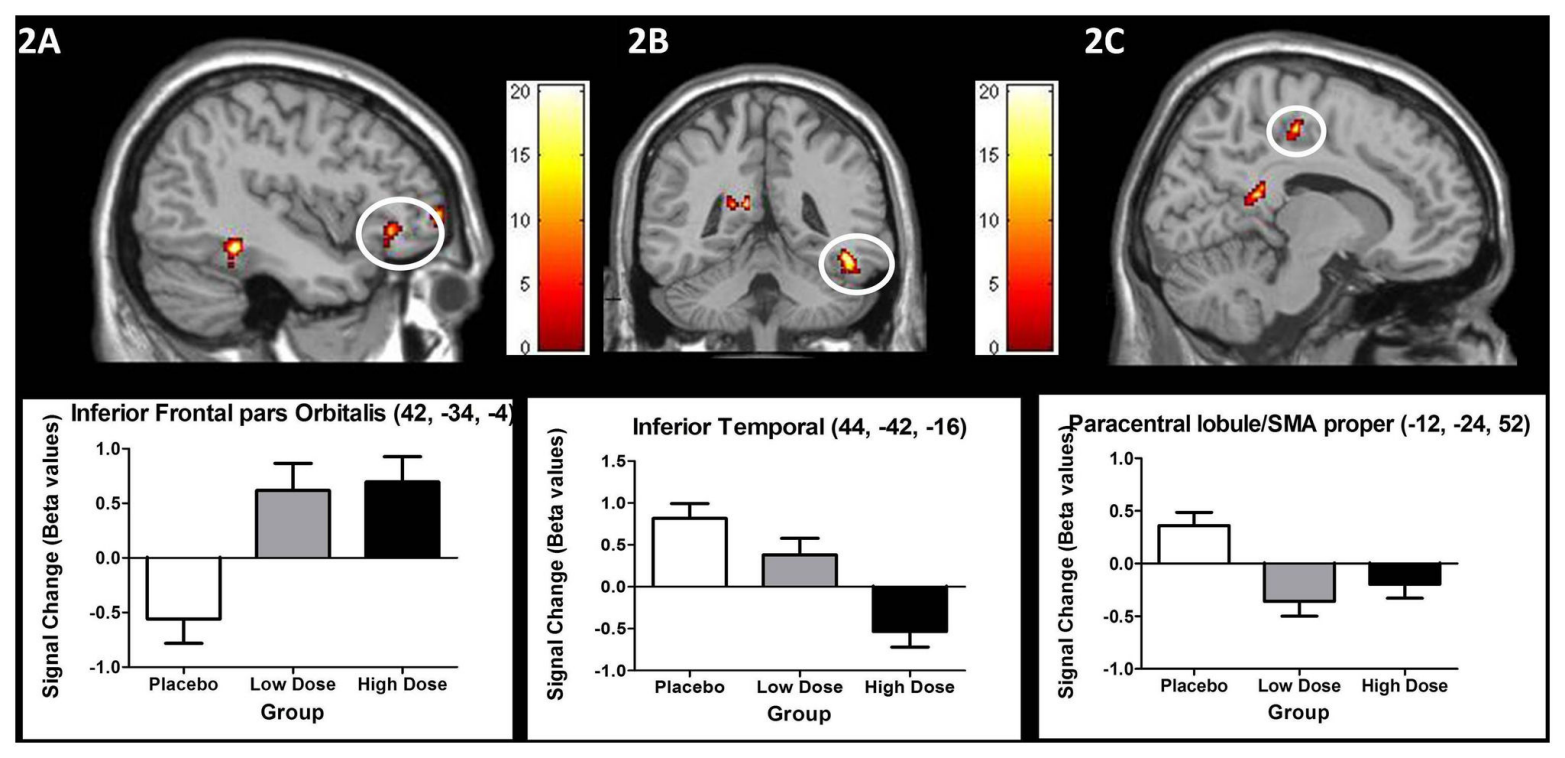
Activations reflecting the main effect of group from the one-way ANOVA Sstop>Go 2^nd^-level model (thresholded at p < 0.005, k = 13; scale represents F statistic; see results section for details). Linear increases were found in inferior frontal gyrus pars orbitalis (Figure 2A), while linear reductions were observed in an inferior temporal cluster (Figure 2B). Non-linear, overall reduction in activation following both doses of alcohol was observed in SMA proper (Figure 2C).

The two doses of alcohol resulted predominantly in non-linear changes in activity (Table 6). Of the regions presented in Table 6, only the inferior frontal pars orbitalis cluster was associated with linear increases in activity as the dose of alcohol increased (Figure 2A), whereas the inferior temporal cluster was associated with linear reductions in activity as the dose of alcohol increased (Figure 2B). Non-linear effects were observed in several key areas (see Table 6) including the paracentral lobule/SMA proper (Figure 2C). These results justify our decision to include planned contrasts between the groups within our analyses.

**Table 6:** Activations resulting from the Sstop>Go contrast One-way ANOVA (p <.005, k ≥ 13 voxels).

**Region**	**Cluster**	**L/R**	**F**	**MNI coord. x, y, z**
***Prefrontal Cortex***				
Middle Frontal gyrus**^*3*^**	20	R	11.09	42 56 2
Inferior Frontal orbitalis (BA47)**^2^**	23	R	9.23	42 34 -4
Paracentral lobule/SMA proper(BA4)**^*3*^**	21	L	8.5	-12-24 52
Paracentral lobule**^*3*^**	13	L	7.11	0 -34 70
***Parietal/Temporal cortex***				
Postcentral/Inferior parietal gyrus**^*3*^**	16	L	7.66	-42-28 34
Posterior cingulate/precuneus**^*3*^**	77	L	11.49	-18-40 16
Inferior Temporal gyrus**^*1*^**	53	R	13.3	44 -42 -16
Parahippocampal gyrus**^*3*^**	18	R	11.71	22-24 -20
Middle Temporal pole/Parahip/pal gyrus**^*1*^**	27	R	9.91	20 4-32

**BA =** **Broadmann** **area;** **R =** **right**, **L** **=** **left** **; MNI coordinates represent cluster peaks; One-** **way** **ANOVA Results: ^*1*^*Linear* decreases;** ^***2***^ **Linear increases;** ^***3***^ **Non-linear* effects*.**

#### Alcohol effects in the VSST: Low dose vs. Placebo: VSST contrast

**Figure 3:**
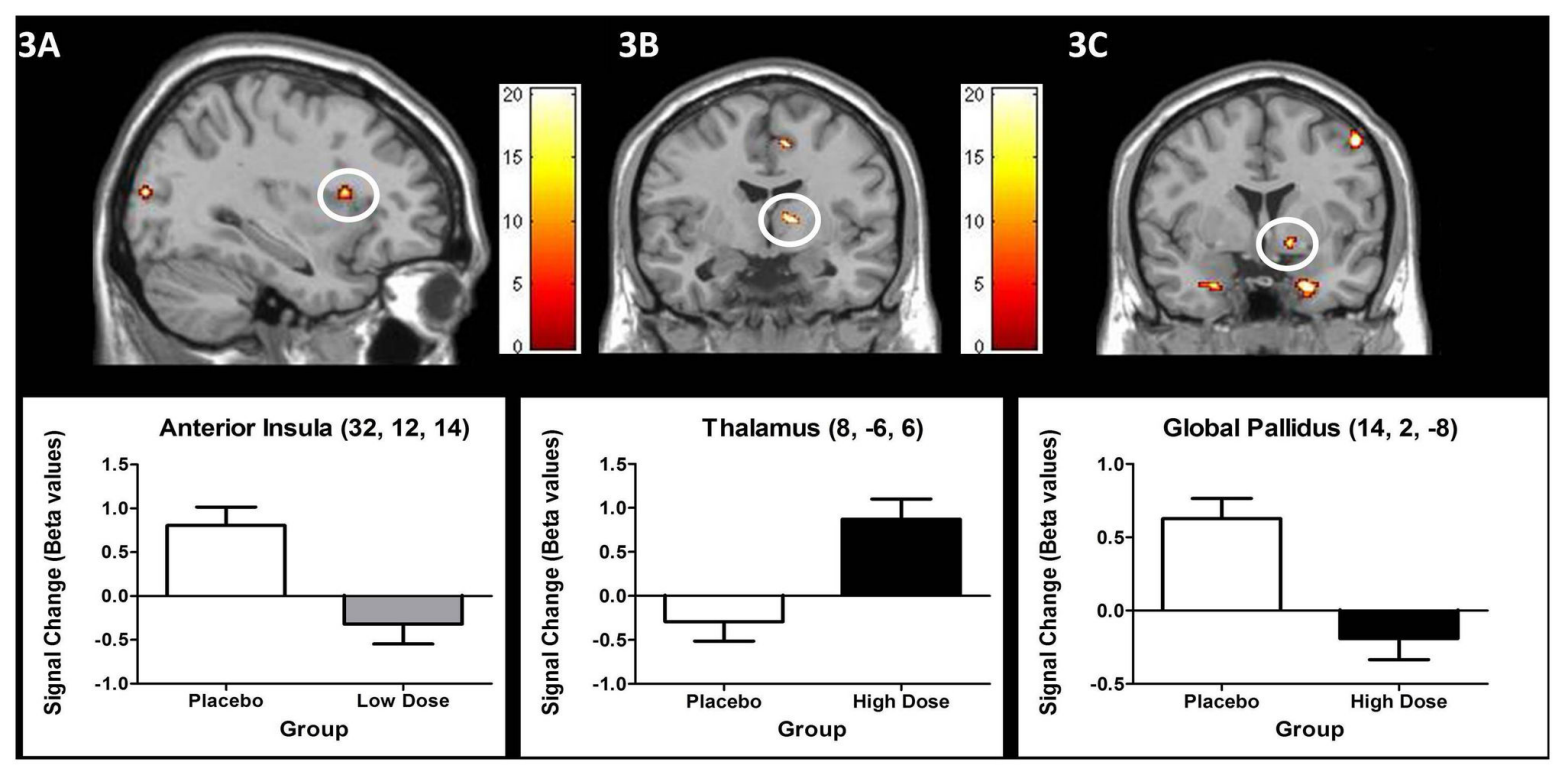
Activations arising from the planned group comparison 2nd- level models (thresholded at p < 0.005, k = 13; scale represents F statistic; see results section for details). Comparison of the Sstop>Go contrast between the low-dose and placebo groups (Figure 3A), and the high-dose and placebo groups (Figure 3B and 3C).

The low dose of alcohol resulted in enhanced responses relative to placebo within parahippocampal and occipital clusters (lingual gyrus, and middle occipital gyrus), as well as in inferior frontal orbitalis, superior frontal gyri and gyrus rectus/ventral anterior cingulate (see Table 7- Low dose vs. Placebo). In contrast, the low dose of alcohol significantly reduced activity in somatosensory and pre-motor cortices, including clusters within the paracentral lobule/SMA proper and postcentral gyrus. The low dose also resulted in decreased activity within anterior insula (Figure 3A) and middle temporal pole.

**Table 7:** Activations resulting from the Sstop>Go contrast group comparisons (p <.005, k ≥ 13 voxels).

**Region**	**Cluster**	**L/R**	**F**	**MNI coord, x, y, z**
***LOW DOSE VS. PLACEBO***				
***Prefrontal Cortex***				
Paracentral lobule/SMA proper (BA4)**^*2*^**	19	L	27.13	-12-24 52
Inferior Frontal orbitalis (BA47)**^*1*^**	18	R	18.58	42 34 -4
Postcentral gyrus**^*2*^**	37	L	14.95	-36-28 36
Anterior Insula**^*2*^**	14	R	12.85	32 12 14
Gyrus rectus/ventral anterior cingulate**^*1*^**	20	R	12.73	10 28 -16
Superior Frontal gyrus(BA9)**^*1*^**	15	L	12.58	-14 48 42
***Parietal/Temporal/Occipital cortex***				
Parahippocampal gyrus **^*1*^**	42	R	29.75	22-24 -20
Middle Temporal pole/Parahip/pal gyrus **^2^**	14	R	17.11	22 4-36
Lingual gyrus**^*1*^**	24	L	15.07	-20-84 4
Middle Occipital gyrus **^*1*^**	23	R	18.58	34-88 14
***HIGH DOSE VS. PLACEBO***				
***Prefrontal Cortex***				
Superior Frontal gyrus (BA32)**^*1*^**	13	R	15.77	16 22 48
Superior Frontal gyrus (BA9)**^*1*^**	18	R	14.89	20 40 48
Inferior Frontal Orbitalis (BA47)**^*1*^**	34	R	17.64	40 32 -6
Middle cingulate/SMA proper (BA6)**^*2*^**	40	R	13.45	10-12 50
Paracentral lobule/SMA proper (BA4)**^*2*^**	18	L	13.73	-12-26 48
Precentral gyrus (BA6)**^*2*^**	23	R	22.17	50 0 50
Postcentral gyrus**^*2*^**	23	L	16.24	-22-44 58
Posterior cingulate/precuneus**^*2*^**	37	L	22.99	-10-44 16
***Parietal/Temporal cortex***				
Inferior Temporal gyrus **^2^**	46	R	26.93	44 -44 -16
Middle Temporal pole**^*2*^**	38	L	20.05	-28 8-36
Middle Temporal pole/Parahip/pal gyrus **^2^**	41	R	21.73	22 4-32
Hippocampus**^*1*^**	13	R	15.11	22 -12 -14
Angular gyrus**^*2*^**	17	L	16.22	-54-66 26
Lingual gyrus (BA18) **^*1*^**	15	L	21.97	-18-74 -8
***Subcortical regions***				
Global pallidus**^*2*^**	13	R	16.92	14 2-8
Thalamus**^*1*^**	13	R	13.48	8 -6 6
***HIGH DOSE VS. LOW DOSE***				
***Prefrontal Cortex***				
Middle Frontal gyrus (BA46)**^*3*^**	29	R	19.7	42 56 2
Inferior Frontal Orbitalis (BA47)**^*4*^**	16	L	17.33	-42 28 -8
Paracentral Lobule (BA4) **^*4*^**	71	R/L	14.69	2-34 70
***Parietal/Temporal cortex***				
Inferior temporal gyrus (BA37) **^*4*^**	20	R	13.88	46 -46 -22

**BA = Broadmann area; R=right, L=left; MNI coordinates represent cluster peaks; Results: ^*1*^*Increased, *^*2*^*Decreased activation relative to placebo; *^*3*^*Increased, *^*4*^*Decreased activation relative to the low dose.***

#### Alcohol effects in the VSST: High dose vs. Placebo: VSST contrast

The high dose of alcohol increased activation in superior frontal and inferior frontal pars orbitalis clusters, as well as in lingual gyrus and in the thalamus (Figure 3B). In contrast, decreased activations were found in premotor cortex that included the SMA. Activity decreases were also observed in output regions of the basal ganglia (global pallidus; see Figure 3C), as well as in clusters within prefrontal cortex and temporal and parietal cortices. Table 7 shows the regions affected by the high dose compared to placebo.

#### Alcohol effects in the VSST: High dose vs. Low dose: VSST_ contrast

The high dose, compared to the low dose of alcohol resulted predominantly in activity reductions (see Table 7 – Low dose vs. High dose). Only the cluster within the right middle frontal gyrus was significantly more activated in the high-dose group relative to the low-dose group.

#### Alcohol effects in the VSST: One-way ANOVA: VSST-Error contrast

The two doses of alcohol resulted predominantly in non-linear changes in activity (Table 8). Of the regions presented in Table 8, only the posterior and middle cingulate clusters were associated with linear increases in activity as the dose of alcohol increased.

**Table 8:** Activations resulting from the Ustop >Sstop Error contrast One-way ANOVA (p <.005, k ≥ 13 voxels).

**Region**	**Cluster**	**L/R**	**F**	**MNI coord. x, y, z**
***Prefrontal Cortex***				
Posterior cingulate^1^	29	L	12.96	-10-42 16
Middle cingulate^1^	14	R	8.16	10-18 46
***Parietal/Temporal cortex***				
Superior Parietal gyrus **^2^**	51	L	14.6	-56-48 12
Parahippocampal gyrus **^2^**	51	R	14.77	22 -22 -24
Inferior Temporal gyrus **^2^**	13	R	7.45	52 -10 -28
Middle Temporal gyrus**^2^**	20	R	12.75	38-58 4
Cuneus**^2^**	14	R	7.63	8-86 34
***Subcortical areas***				
Caudate**^2^**	20	L	11.68	-14 16 16

**BA = Broadmann area; R = right, L = left; MNI coordinates represent cluster peaks; Results: ^*1*^*Linear increases; *^*2*^*Non-linear effects***

*Low dose vs. Placebo: VSST-Error contrast:* The low dose of alcohol enhanced responses relative to placebo within the paracentral lobule, the middle temporal gyrus and the caudate. Reduced activity was found in other clusters within the temporal cortex, and in the thalamus (see Table 9 – Low dose vs. Placebo).

**Table 9:** Activations resulting from the Ustop> Sstop contrast group comparisons (p <.005, k ≥ 13 voxels).

**Region**	**Cluster**	**L/R**	**F**	**MNI coord, x, y, z**
***LOW DOSE VS. PLACEBO***				
***Prefrontal Cortex***				
Paracentral lobule**^*1*^**	91	L	13.28	-12-26 52
Paracentral lobule**^*1*^**	41	R	18.88	12-36 56
***Temporal/Cerebellum***				
Superior temporal gyrus**^*2*^**	21	R	15.38	38 2-16
Middle Temporal**^*1*^**	30	R	26.3	38-58 2
Middle Temporal Pole**^*2*^**	22	L	20.13	-24 0 -36
Parahippocampal gyrus**^*2*^**	69	R	24.95	22 -22 -22
Fusiform**^*2*^**	29	R	13.7	40 -40 -18
Cerebellum**^*2*^**	17	R/L	16.75	10-58 -10 (-14-72 -16)
***Subcortical areas***				
Caudate**^*1*^**	39	L	21.64	-14 18 18
Thalamus**^*2*^**	15	R	11.67	8-28 6
***HIGH DOSE VS. PLACEBO***				
***Prefrontal Cortex***				
Posterior Cingulate**^*1*^**	50	L	28.38	-10-42 16
Middle cingulate**^*1*^**	54	R	16.36	10-16 46
Ventral Anterior cingulate**^*1*^**	18	L	19.95	-10 34 -8
Rostral Orbitofrontal (BA11) **^*2*^**	13	R	16.15	10 54 -20
Superior Frontal gyrus (BA9) **^*2*^**	21	R	15.25	10 44 46
Precentral gyrus**^*1*^**	24	R	14.85	16-22 58
Precentral gyrus**^*1*^**	13	R	13.69	30 -6 46
Postcentral gyrus**^*1*^**	15	R	17.24	24-36 48
***Temporal cortex***				
Parahippocampal gyrus**^*2*^**	41	R	25.53	24 -22 -22
Inferior temporal gyrus**^*1*^**	32	R	22.14	44-46 -14
***Subcortical regions***				
Thalamus**^*2*^**	21	R	15.84	6-8 18
***HIGH DOSE VS. LOW***				
***Prefrontal Cortex***				
Middle frontal gyrus**^*4*^**	13	R	19.27	36 62 2
Paracentral Lobule**^*3*^**	38	R	18.61	2-32 72
Postcentral gyrus**^*3*^**	31	R	15.66	42 -26 56
***Parietal/Temporal cortex***				
Superior Parietal**^*3*^**	43	L	16.84	-22-52 56
Cuneus**^*3*^**	32	L	14.65	-18-70 38
Inferior temporal**^*3*^**	66	R	19.2	46 -44 -22
Parahippocampal gyrus**^*3*^**	13	R	12.6	18 -6 -22
Cerebellum**^*3*^**	21	R	18.94	38-66 -26 (12-38 -22/38 -64-40)

**BA = Broadmann area; R = right, L = left; MNI coordinates represent cluster peaks; Results: ^1^*Increased*, ^*2*^Decreased activation relative to placebo; ^*3*^*Increased*, ^*4*^*Decreasedactivationrelativetothelowdose*.**

#### Alcohol effects in the VSST: High dose vs. Placebo: VSST-Error contrast

The high dose of alcohol increased activation in clusters within cingulate cortex (ventral ACC, middle and posterior), somatosensory and motor cortices (precentral and postcentral gyrus) and in the inferior temporal gyrus. By contrast, decreased activations were found in the parahippocampal gyrus, in frontal clusters (the superior frontal gyrus and a rostro medial cluster of the orbitofrontal cortex), and in the thalamus (see Table 9 – High dose vs. Placebo).

#### Alcohol effects in the VSST: High dose vs. Low dose: VSST-Error contrast

The high dose, compared to the low dose of alcohol resulted predominantly in activity increases (see Table 9 – Low dose vs. High dose). Only a cluster within the right middle frontal gyrus was significantly less activated in the high-dose group relative to the low-dose group.

#### Regions of interest analysis: VSST contrast

Within ROIs defined by the conjunction analysis between VSST and VSST_C (Table 5), there was no significant main effect of group, indicating that alcohol did not affect performance on the VSST via an effect on regions commonly activated during the processing of infrequent, visually salient Stop-signals.

#### Results from one-way ANOVA in the control task

Alcohol-related changes in activation associated with the control contrast (i.e. Stop_VSST_C> Go_VSST_C) were observed within prefrontal cortex, parietal, temporal and occipital cortex as well as thalamus and cerebellum (Table 10). These observations suggest that the effects of alcohol extend to the processing of task-irrelevant Stop-signals.

**Table 10:** Activations resulting from the Stop_VSST_C>Go_VSST_C contrast One-way ANOVA (p <.005, k ≥ 13 voxels).

**Region**	**Cluster**	**L/R**	**F**	**MNI coord. x, y, z**
***Prefrontal Cortex***				
Medial superior frontal**^2^**	14	L	9.87	-12 56 12
Anterior cingulate**^2^**	22	R	9.72	2 12 30
Inferior frontal tri (BA45) **^2^**	16	R	9.48	58 26 20
***Parietal/Temporal cortex***				
Mid temporal gyrus **^1^**	51	L	14.6	-56-48 12
Inferior Temporal gyrus **^2^**	43	L	14.2	-58-18 -24
Inferior Temporal gyrus **^2^**	13	R	7.45	52 -10 -28
Superior temporal pole**^2^**	34	R	10.4	40 20 -18
Superior temporal pole**^2^**	37	L	9.16	-30 12-22
Superior parietal gyrus **^2^**	26	L	8.36	-28-58 52
Cuneus**^2^**	14	R	7.63	8-86 34
***Occipital cortex/Cerebellum***				
Calcarine**^2^**	13	L	8.89	-12-70 14
Mid occipital gyrus **^2^**	43	L	10.1	-42-76 18
Cerebellum **^2^**	66	R	12.5	18-56 -14
Cerebellum^1^	38	L	11.87	-10-78 -50
***Subcortical areas***				
Thalamus**^2^**	13	L	10.83	-18-30 8

**BA = Broadmann area; R = right, L = left; MNI coordinates represent cluster peaks; Results: ^*1*^*Linear decreases; *^*2*^*Non-linear effects***

## Discussion

The present study assessed the effects of two acute doses of alcohol on the inhibition of an initiated motoric response, and explored the brain mechanisms associated with these effects. Two versions of a visual Stop-signal task were used. One involved stopping an initiated response at the onset of a Stop-signal, while the other required participants to respond regardless of whether the Stop-signal appeared or not. This latter condition challenged perceptual and attentional processes associated with the presentation of infrequent Stop-signals, yet was uncontaminated by processes of response inhibition.

Behaviourally, acute alcohol at both the high, and the low dose significantly impaired inhibitory control, relative to placebo, as evidenced by a significant increase in SSRT in the typical version of the task. Importantly, we titrated the task to maintain the same level of performance accuracy, thus the effects of alcohol were restricted to inhibitory processes, consistent with previous data [6,19]. In the present study, attentional processes remained unaffected by alcohol at the doses used here: alcohol did not affect accuracy or reaction time on any trial-type in the control variant of the VSST, in which inhibition of a behavioural response was not required. In a previous study, an effect of alcohol was found on attention-dependent control of interference from task irrelevant stimuli [68].

The VSST was successful at producing measurable changes in BOLD responses in regions previously implicated in response inhibition. These included regions within the inferior frontal and dorsolateral prefrontal cortex (e.g. middle frontal gyrus), the right insular cortex, the pre-SMA, and the basal ganglia [23,24,25,41]. Moreover, we demonstrated (by exclusive masking with the control variant of the VSST) that motor and pre-motor regions (pre-SMA; SMA proper and precentral cortex), dorsolateral PFC (middle frontal gyrus), insular cortex and clusters within the basal ganglia (putamen and caudate) were selectively involved in successful stopping, consistent with recent studies [35,39]. These regions are encompassed within proposed fronto-basal ganglia-thalamo-cortical ‘loops’ implicated in the control of programmed motoric functions [28]. By contrast, conjunction analyses with the control contrast demonstrated that some regions were not uniquely involved in successful stopping, but were also sensitive to the processing of infrequent perceptually-salient cues. These included the right inferior frontal gyrus pars opercularis and areas within right parietal and temporal cortices [35,39].

Interestingly, the effects of the two alcohol doses on activations associated with successful stopping, appear to be expressed regionally differentiated. Linear reductions in activation, as the dose of alcohol increased, were observed within the inferior temporal cortex. Non-linear changes were observed in clusters within PFC, including middle frontal cortex and paracentral lobule/SMA proper, regions that have been implicated in successful stopping [23,24,25]. Importantly, the activity we observed within the paracentral lobule/SMA proper cluster was selective to successful stopping and was not sensitive to the perceptual attributes of the visual cues, as it remained unaffected by exclusive masking with the control contrast. Thus changes in activation under alcohol were seen in this region only when inhibition of an initiated motoric response was required. Non-linear changes in activation were also seen in parietal and temporal cortex, including regions associated with stimulus-representation [69]. Linear increases in activation were observed within the right Inferior frontal gyrus pars orbitalis, an area involved in the modulation of inhibitory responses [70].

During successful stopping the low dose of alcoholevoked both increases and decreases in activation, relative to placebo, within a set of cortical regions associated with integrative sensorimotor and cognitive processes including parietal, temporal, occipital and prefrontal regions. Particularly noteworthy is the engagement of anterior insular cortex, where activity, reduced by alcohol, was related to successful stopping (not the processing of infrequent cues). This observation is interesting from the perspective of what is known about anterior insula engagement in the representation of emotional and motivationally- salient stimuli [71]. In the context of addiction and appetitive behaviours, the anterior insula is proposed as an interface between embodied internal signals and their expression in motor urges, rituals, and affective states [72]. Interestingly, Anderson and colleagues [42] identified a neighbouring area of insula in which activity was affected by a high dose of alcohol during unsuccessful no-go trials in a Go/No-Go task.

The high alcohol dose evoked changes in activity in several of those areas affected by the low dose, including paracentral lobule/SMA proper, but importantly induced changes in activity within subcortical centres. It enhanced activation, relative to the placebo group, in the thalamus, but it decreased it in the global pallidus and in the paracentral lobule/SMA proper. The global pallidus is known to suppress the thalamic “go” output to motor cortices in order for an initiated motoric response to be terminated [23,25,29,30,31]. The activation changes seen in the present study within these areas (global pallidus and thalamus) and the absence of observed activity changes within the subthalamic nucleus suggests that only the indirect pathway was affected by a high alcohol dose. However, our fMRI study was conducted at a field strength of 1.5 T, where technical limits on sensitivity may also account for why we may not have detected activation changes within the subthalamic nucleus. Our observations suggest this subcortical control mechanism is disrupted by higher doses of alcohol, yet further highlight the parallel role of SMA activity in motor response inhibition and its sensitivity to effects of alcohol (previously recognised in the performance of a Go/No-Go task) [42].

Our findings also highlight a functional distinction in the role of the anterior cingulate cortex. Activation within the anterior cingulate was affected in a non-linear way by the two doses of alcohol, but only in the VSST_C contrast (Stop_VSST_C> Go_VSST_C). Previous studies looking at the effects of alcohol on interference tasks also report modulation of activity within this region by alcohol [68,73]. These observations suggest that alcohol compromises processes engaged in the suppression of interference from task-irrelevant Stop-signals (the red arrow in the VSST_C).

The dorsal anterior cingulate cortex has also been discussed in the literature as an area affected by a high dose of alcohol in conjunction with error processing [42]. However in the present study, when we examined unsuccessful versus successful stop trials, we only found activation under alcohol (high dose) in subregions of cingulate cortex (middle and posterior) associated with other functions including attention and working memory [74]. It is worth to note that, due to the implementation of the staircase procedure in the SST, participants had to adjust their performance so that error monitoring probably occurred throughout the task. This was not the case in the study by Anderson and colleagues [42] in which participants adjusted their performance only under instruction and at an early stage of the task.

Taken together, our data suggest that whereas both the high and low alcohol dose may modulate brain activation in regions involved in ‘representational’ sensorimotor and motor planning processes, only the high dose of alcohol modulates subcortical areas of the fronto-striatal-thalamo-cortical loops, specifically globus pallidus and thalamus, that are arguably more proximate to the final motor response.

It is noteworthy that in this study, neither dose of alcohol was found to affect the activity of regions identified in the conjunction analysis of the typical VSST and control tasks, i.e. brain areas engaged by the perceptual processing of infrequent, visually salient signals. This suggests that alcohol does not affect VSST performance through effects expressed at the level of visual sensory representations. Nevertheless, alcohol may still produce deficits in the capacity to shift attention, or potentially reduce overall cognitive/attentional set. This may account in part for the group effects of alcohol observed in the control task on activity within temporal and parietal regions associated with visual attention.

Acute alcohol, relative to placebo, can increase cerebral blood-flow in lateral prefrontal, as well as in medial frontal regions [75,76]. Given that we infer task-induced changes in regional neural activity from haemodynamic changes (i.e. the BOLD signal) [77], it is possible that alcohol’s general vasoactive effects may interact with activity patterns attributed to task effects. Arterial spin labeling, which is a direct measure of cerebral perfusion, may prove a useful tool for future studies for disambiguating the potential vascular effects of acute alcohol from its effects on neuronal activity. Nevertheless, in this study, we observed both increases and decreases in task-related BOLD response associated with alcohol ingestion at different doses. Moreover distributed regions (e.g. those identified in the conjunction analyses) were not modulated by alcohol. Consequently, the absence of global, and the emphasis on short term, changes in signal (apparent after high-pass temporal-filtering) mitigate the potential confounding impact of alcohol-induced vasoactive-related changes on our findings. While the present study used a between-subjects design for the administration of alcohol, which may have introduced inter-subject variability to both the behavioural and functional data, participants in each group were well matched on all baseline measures, including those most relevant to the experimental task, such as trait impulsivity and baseline stop-signal task performance. Future studies could benefit from within-subject alcohol administration, as long as behavioural and neuronal effects of repetition are properly controlled for. An important limitation for our major conclusion is that, because the VSST_C task always followed the inhibitory VSST, participants might still have engaged inhibitory control processes to inhibit the previous learned stop response that was no longer required in the control task. However, our data suggest this possibility was not a major confound: If there was an inhibitory component evoked during the control task, the Go reaction time should have been equally long in the two tasks (VSST_C and VSST). However, Go reaction time was significantly faster in the VSST_C than in the VSST. Nevertheless, to clarify this limitation, future studies should administer the two tasks in a counterbalanced order.

In summary, the present study provides evidence that alcohol at low and high doses affects motor inhibitory control by modulating activation within prefrontal, parietal, temporal and motor cortical areas. Moreover, the high dose of alcohol additionally affects subcortical nodes within fronto-basal ganglia- thalamo-cortical motor loops known to be involved in initiating and terminating a motoric response. Alcohol had little effect on regions associated with the perceptual processing of an infrequent cue. These findings detail the processes and the underlying neural substrates through which alcohol impairs behavioural control as expressed in motor inhibition, and are relevant to understanding the progression of deficits in self-regulation and inhibition in problem drinking with implications for addictive disorders.
